# Enhanced Antibacterial Activity of *Artemisia absinthium* Extract Containing Artemisinin and Polyphenols Loaded into Mesoporous Silica Calcium- and Cerium-Doped Nanoparticles

**DOI:** 10.3390/jfb17070326

**Published:** 2026-07-06

**Authors:** Ioannis Tsamesidis, Georgia K. Pouroutzidou, Athanasios Christodoulou, Dimitrios Gkiliopoulos, Dionysia Amanatidou, Styliani Axypolitou, Maria Bousnaki, Georgia Michailidou, Dimitrios Bikiaris, Phaedra Eleftheriou, Maria Chatzidimitriou, Sotirios Kalfas, Eleana Kontonasaki

**Affiliations:** 1 Department of Prosthodontics, Faculty of Health Sciences, School of Dentistry, Aristotle University of Thessaloniki, 54124 Thessaloniki, Greece; gpourout@physics.auth.gr (G.K.P.); thanosxristodoulou5@gmail.com (A.C.); stella.ax2@gmail.com (S.A.); mbousnaki2689@hotmail.gr (M.B.);; 2 Department of Biomedical Sciences, Faculty of Health Sciences, International Hellenic University, Sindos, 57400 Thessaloniki, Greece; damanatidou@ihu.gr (D.A.); elfther@ihu.gr (P.E.); mchatzid952@gmail.com (M.C.); 3 Laboratory of Chemical and Environmental Technology, Department of Chemistry, Aristotle University of Thessaloniki, 54124 Thessaloniki, Greece; dgiliopo@chem.auth.gr; 4 Laboratory of Polymer Chemistry and Technology, Department of Chemistry, Aristotle University of Thessaloniki, 54124 Thessaloniki, Greecedbic@chem.auth.gr (D.B.); 5 Department of Preventive Dentistry, Periodontology & Implant Biology, Dental School, Aristotle University of Thessaloniki, 54124 Thessaloniki, Greece; kalfas@dent.auth.gr

**Keywords:** *Artemisia absinthium*, artemisinin, mesoporous nanoparticles, antibacterial properties, reactive oxygen species, total antioxidant activity

## Abstract

Background: *Artemisia absinthium* (*A. absinthium*) is a perennial plant valued for its antibacterial, antioxidant, and anti-inflammatory properties, exhibiting broader therapeutic potential. Given the need to deliver low doses of *A. absinthium* extract, mesoporous silica nanoparticles have attracted considerable attention as promising nanocarriers due to their distinctive physical and chemical properties. Methods: Physicochemical characterization of the materials was performed and biological assays were conducted to investigate the ROS, antibacterial and antioxidant activity of *A. absinthium* extract encapsulated within cerium- and calcium-doped mesoporous silica nanoparticles (MNSiCaCe) against both aerobic and anaerobic bacteria. Results: FTIR, SEM, and BET analysis confirmed successful synthesis of the MNSiCaCe. Phytochemical profiling of *Artemisia absinthium* extract using HPLC revealed the presence of artemisinin and a rich composition of phenolic and flavonoid constituents, with a total phenolic content of 182 ± 3.6 mg GAE/100 g dry plant material and a total flavonoid content of 42.5 ± 0.6 mg QE/100 g. Quantitative drug loading profiling demonstrated that while plain MNSi nanocarriers achieved a loading capacity of 16.96%, the MNSiCaCe enhanced this threshold to 43.11%. The in vitro controlled-release kinetics exhibited a highly prolonged and slow-release profile of the MNSiCaCe. The materials demonstrated excellent hemocompatibility and high mitochondrial activity with human periodontal ligament cells (hPDLCs). Elevated ROS generation was observed under conditions where antibacterial activity was most pronounced. While the artemisinin-doped nanoparticles showed notable antibacterial effects, the complete *Artemisia absinthium*-loaded nanoparticles achieved a significantly greater reduction in bacterial viability probably due to the synergistic interaction between artemisinin and the extract’s rich polyphenol profile. Conclusions: These findings highlight MNSiCaCe as a promising and safe nanocarrier system for drug delivery, with strong antibacterial potential, offering valuable applications in antibacterial therapies.

## 1. Introduction

In recent years, green chemistry has received increasing attention as an environmentally friendly and sustainable methodology for synthesizing active pharmaceutical ingredients. *Artemisia absinthium* (*A. absinthium*), commonly known as absinthe or wormwood, is a perennial plant characterized by broad ovate pointed leaves of a silver-gray coloration and can grow up to 1.5 m in height. The essential oils of *A. absinthium* contain several key components, including thujone, thiols, bitter substances, absinthin, artabsin, artemisinin, anabsinthin, flavonoids, ascorbic acid, and tannins [1]. The earliest reference to absinthe, reportedly used for disinfection in religious rituals, appears in an Egyptian papyrus dating to approximately 1600 BC. The ancient Romans employed *A. absinthium* to treat seasickness, whereas during the Middle Ages it was commonly cultivated in monastery gardens and valued for its medicinal properties. It later became a key ingredient in the well-known liqueur “absinthe”. However, excessive consumption of absinthe can lead to neurotoxic effects due to thujone, its principal active compound, which is known to cause hallucinations and central nervous system disturbances. One of the most famous alleged victims of absinthe intoxication was the artist, Vincent van Gogh. The production and sale of absinthe liqueur were eventually banned in numerous countries [2].

Despite its risks in high doses, *A. absinthium* possesses several therapeutic properties. Absinthe infusions are traditionally used to stimulate appetite, relieve bloating and colic, and alleviate gastric discomfort. Due to its intense bitterness, its application as a culinary herb is limited, although small quantities of its aromatic leaves are sufficient to enhance the flavor of various meat dishes. Additionally, the plant exhibits uterotonic properties; in certain Greek villages, it has traditionally been administered during labor to aid uterine contractions.

The silver-gray leaves of *A. absinthium*, which are highly aromatic, are typically harvested before the flowering stage and can be used either fresh or dried in powdered form to exert anthelmintic and anti-inflammatory effects [3]. *A. absinthium* has been widely employed in both traditional and modern medicine for the treatment and prevention of fevers, chills, and malaria [4]. Extracts from this medicinal plant are recognized for their notable antioxidant [5] and antibacterial [6] activities. One of the key bioactive constituents responsible for these therapeutic properties is artemisinin (ART). The concentration of artemisinin in *A. absinthium* extracts varies depending on the plant variety and the harvesting season (e.g., autumn vs. summer) [7]. ART is a potent antimalarial compound known for its rapid activity against Plasmodium parasites in the bloodstream. Discovered in the 1970s by Chinese scientist Tu Youyou, this compound significantly advanced malaria treatment and led to her receiving the Nobel Prize in Physiology or Medicine in 2015 [8].

As an antimalarial agent, ART is considered the first-line treatment for Plasmodium falciparum malaria due to its rapid onset of action, ability to target multiple stages of the parasite’s life cycle, including both asexual and sexual blood stages and its favorable safety profile [9]. Consequently, many malaria-endemic countries have adopted ART-based combination therapy (ACT) as the standard treatment for uncomplicated malaria.

Furthermore, ART has demonstrated antibacterial effects against a variety of bacterial species, exhibiting antimicrobial efficacy comparable to that of the antibiotic streptomycin [10]. As an antiviral and antifungal agent, ART has been evaluated against several viruses, including human cytomegalovirus, herpesviruses, hepatitis B and C viruses, and human immunodeficiency virus, showing promising antiviral potential [11]. Although its clinical use for viral infections remains under investigation, evidence suggests that ART can inhibit the replication of various viruses, such as flaviviruses. Regarding its antifungal activity, ART has shown some efficacy; however, it is not commonly employed as a treatment for fungal infections [12]. Moreover, emerging evidence indicates that artemisinin exhibits not only strong anti-inflammatory and immunomodulatory properties, but also significant neuroprotective effects. These include potential therapeutic benefits against age-related macular degeneration and Alzheimer’s disease, as demonstrated in cellular and animal models [13].

Given the need to deliver low doses of *A. absinthium* extract effectively to human cells, the development of efficient delivery vehicles is essential. In this context, mesoporous silica nanoparticles (MSNs) have attracted considerable attention as promising nanocarriers due to their distinctive physical and chemical properties. Their high surface area, tunable pore size, and modifiable surface chemistry make them particularly well-suited for encapsulating and delivering bioactive compounds such as *A. absinthium* extract with enhanced precision and stability. The highly ordered pore structure and customizable surface features of MSNs further support their suitability for a wide range of biomedical applications [14].

Incorporating calcium ions into these nanoparticles has been shown to significantly enhance their biological performance. This modification facilitates the controlled release of calcium ions, which can positively influence interactions with both bone tissue and human cells [15]. Calcium doping improves bioactivity, enhances compatibility with physiological environments, and promotes mineralization, an essential process in tissue regeneration. Consequently, calcium-doped mesoporous silica nanoparticles (Ca-MSNs) have emerged as promising platforms for targeted drug delivery, tissue engineering, and dental applications [16].

**Figure 1:**
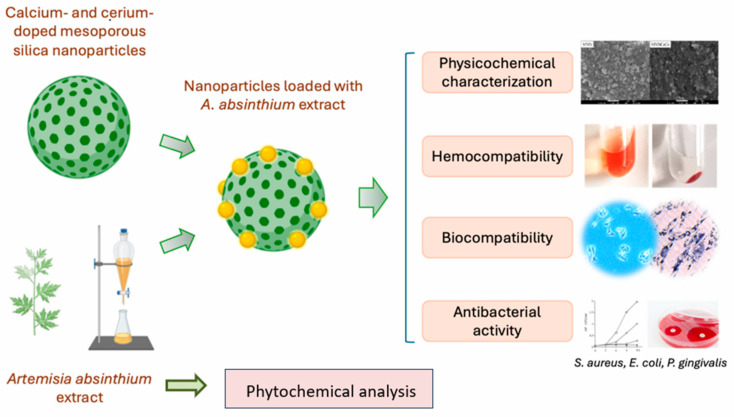
Graphical illustration of the study.

In the present study, *A. absinthium* extract was loaded into mesoporous silica nanoparticles co-doped with calcium and cerium ions, at hemocompatible concentrations, to assess their antibacterial activity, as well as their effects on human periodontal ligament cells (Figure 1).

## 2. Materials and Methods

### 2.1. A. absinthium Extract Preparation and Phytochemical Profiling

Leaf samples of *A. absinthium* were acquired from an accredited grower in Ilioupoli, Thessaloniki, and were originally collected in June 2023 from Crete, Greece. Their identity was formally authenticated by a botanist from the Department of Chemistry and Pharmacy at Aristotle University of Thessaloniki, following a detailed evaluation of the plant’s morphology, habitat, and organoleptic characteristics. For internal batch traceability, the producer assigned a unique coding system to the leaves. The specimens were collected on 14 June 2023, from Crete, Greece (GPS coordinates: 35.417416° N, 24.530005° E), and are cataloged under voucher number 125. The *A. absinthium* extract was prepared as previously described [17]. The extract was analyzed to determine its total polyphenol content (TPC) and total flavonoid content (TFC) [18]. TPC was expressed as milligrams of gallic acid equivalents per gram of dry plant material (mg GAE/g dry plant), whereas TFC was presented as milligrams of quercetin equivalents per 100 g of dry plant material (mg QE/100 g dry plant).

For artemisinin identification, quantitative analysis was performed using a Shimadzu HPLC (Kyoto, Japan) prominence system consisting of a degasser (DGU-20A5, Kyoto, Japan), a liquid chromatograph (LC-20 AD, Kyoto, Japan), an autosampler (SIL-20AC, Kyoto, Japan), a UV/Vis detector (SPD-20A, Kyoto, Japan), and a column oven (CTO-20AC, Kyoto, Japan). For the analysis, the well-established method of Liu et al. was carried out: CNW Technologies Athena C18, 120 A, 5 μm, 250 mm × 4.6 mm at a column temperature of 30 °C. The mobile phase consisted of H_2_O/ACN 40/60 *v*/*v*, at a flow rate of 1.0 mL/min. The injection volume was 20 μL and UV detection was performed at 210 nm at 30 °C. The other bioactive compounds present in the *A. absinthium* extract were separated using HPLC using Agilent Technologies 1260 Infinity HPLC system and a ReproSil Gold 120 C18 column (25 cm length, 4.6 mm internal diameter, and 5 μm particle size) (Kyoto, Japan).

The mobile phase consisted of an aqueous solution containing 2% acetic acid (A) and methanol (B) with a linear increase in methanol concentration in the mixture as follows: Isocratic solution containing 5% of B for 2 min followed by a gradual increase (gradient) of B concentration to 25% at 10 min and to 40%, 50% and 60% every 10 min; as described by Boligon et al. [18]. The temperature was maintained at 25 °C throughout the analysis. The flow rate was 0.7 mL/min and the injection volume was 20 μL. Quantification was carried out by integration of the peaks using the external standard method, at, 257 nm for gallic acid, 325 nm for methyl caffeate, and 365 nm for quercetin and astragalin (kaempferol-3-glucoside).

### 2.2. Synthesis of Silica-Based Mesoporous Nanoparticles

Mesoporous silica (MNSi) and Ca- and Ce-doped silicate nanoparticles (MNSiCaCe) were synthesized under alkaline conditions (final solution pH 12) using the sol–gel method, as described in the literature, with some modifications using cetyltrimethylammonium bromide (CTAB) as pore-forming agent for the mesoporous structure. The reactants were sodium hydroxide (NaOH, alkaline medium), CTAB, tetraethyl orthosilicate (TEOS), Ca(NO_3_)24H_2_O, and Ce(NO_3_)_3_6H_2_O from Merck KGaA, Darmstadt, Germany. The synthesized materials were dried at 60 °C overnight and underwent calcination at 600 °C for 5 h to remove the pore directing agent. The nominal compositions of the produced nanoparticles are shown in Table 1 below.

### 2.3. Preparation of Artemisia absinthium-Loaded Nanoparticles, Loading Capacity and Controlled-Release Kinetics of Modified Mesoporous Silica Nanoparticles

To prepare the loaded nanoparticles, *Artemisia absinthium* (AE) extract (ΕΧ) was incorporated into 10 mL of an alkynylated mesoporous nanoparticle suspension (1 mg/mL) and stirred at 300 rpm for 24 h at room temperature under dark conditions. The resulting AE-loaded nanoparticles were isolated via centrifugation at 5000× *g* for 15 min and subsequently dried at ambient temperature. Quantitative analysis of the remaining supernatant was performed using a Shimadzu Prominence HPLC system equipped with a DGU-20A5 degasser, LC-20AD liquid chromatograph, SIL-20AC autosampler, SPD-20A UV/Vis detector, and a CTO-20AC column oven. The drug loading capacity (LC) was determined using the formula: Loading capacity = [(Total amount of ART)/ART Loaded nanoparticles weight] × 100. For in vitro release profiles, 10 mL of an alkynylated mesoporous nanoparticle suspension (1 mg/mL) in phosphate-buffered saline (PBS) at pH 7.4 was incubated at room temperature under constant agitation at 300 rpm. At scheduled intervals (0, 6, 17, 21, 24, 41, 48, 72, 96, 120, and 240 h), supernatants were collected by removing a sampling volume of 0.5 mL, which was immediately replaced with an equal volume of fresh, pre-warmed PBS to maintain sink conditions. The collected samples were appropriately stored and quantified for artemisinin content within the extract, with all experimental measurements performed in triplicate.

### 2.4. Physicochemical Characterization

Fourier-Transform Infrared Spectroscopy (FTIR). Nanoparticles were characterized using a Perkin Elmer spectrometer (Perkin Elmer Inc., Waltham, MA, USA), in transmission mode (400–4000 cm^−1^), using a resolution of 2 cm^−1^ and 32 scans. KBr pellets (Merck KGaA, Darmstadt, Germany) were prepared under a pressure of 7 tons using a sample-to-KBr mass ratio of 1:100.

Brunauer–Emmett–Teller (BET) and Brunauer–Joyner–Halenda (BJH) analyses. The mesoporous structure of the nanoparticles was characterized by N2 adsorption–desorption (Autosorb-1MP, Quantachrome Instruments, Boynton Beach, FL, USA) at −196 °C using the Brunauer–Emmett–Teller (BET) and Brunauer–Joyner–Halenda (BJH) methods.

Z-potential. The zeta potential of the samples was measured using a Litesizer 500 instrument (Anton Paar, Graz, Austria). Prior to analysis, the samples were dispersed in water at a concentration of 100 ppm and ultrasonicated for 5 min to ensure proper dispersion.

### 2.5. Scanning Electron Microscopy and Energy Dispersive Spectroscopic Analysis (SEM-EDS)

The morphology and elemental composition of the materials were analyzed using a JEOL JSM-7610F Plus field-emission scanning electron microscope, with samples prepared by depositing a small amount of powder onto conductive adhesive tape and subsequently coating them with a 200 Å thick carbon layer to enhance electrical conductivity prior to imaging.

### 2.6. Biological Assays

MTT assay. Cell viability was assessed by incubating the materials with human periodontal ligament cells (hPDLCs), which were isolated from healthy donor biopsy samples obtained during routine third molar extractions and subsequently cultured, as previously described [19]. The metabolic activity of hPDLCs exposed to the test materials was evaluated on days 1 and 3 of culture. Cells at passages 2–4 were seeded into 96-well plates at a density of 1 × 10^4^ cells/well and allowed to adhere for 24 h. Subsequently, the cells were exposed to two concentrations (0.125 and 0.5 mg/mL) of either plain nanoparticles or *Artemisia* extract-loaded nanoparticles. All experiments were performed in quintuplicate.

Mitochondrial activity as an indicator of cell metabolism and proliferation, was assessed using the MTT (3-(4,5-dimethylthiazolyl-2)-2,5-diphenyltetrazolium bromide) assay as previously described [20].

Hemocompatibility assay. The hemocompatibility assay was performed using the same experimental setup and material groups as those used for the MTT assay. The plain and extract-loaded nanoparticles were evaluated by assessing erythrocyte lysis. The assay was performed in quintuplicate by incubating the nanoparticles with human erythrocytes for 24 h at 37 °C at a fixed hematocrit of 2%. Untreated erythrocytes served as the negative control, while hemolysis induced with distilled water served as the positive control. After incubation, samples were centrifuged at 3000× *g* rpm for 5 min, and the absorbance of released hemoglobin in the supernatant was measured at 541 nm with 700 nm as the reference wavelength, as previously described [21].

Antibacterial assay. The strains of *Staphylococcus aureus* and *Escherichia coli* used in this study were clinical isolates kindly provided by Prof. Maria Chatzidimitriou. These aerobic clinical isolates were previously isolated from clinical samples and identified using standard morphological and biochemical criteria according to Cowan and Steel’s manual. The antibacterial activity of the nanoparticles was examined against two aerobic clinical isolates: *Staphylococcus aureus* (*S. aureus*) and *Escherichia coli* (*E.coli*). The isolates were identified using standard biochemical tests (Cowman & Steel). Pre-weighed amounts of nanoparticles were sterilized by UV light exposure and suspended in TSY broth (tryptic soy broth supplemented with 0.5% yeast extract) in sterile tubes to achieve final nanoparticle concentrations of 0.125 mg/mL and 0.5 mg/mL. To standardize the inoculum according to CLSI M07 guidelines, overnight bacterial cultures were adjusted to match a 0.5 McFarland turbidity standard (1 × 10^8^ CFU/mL). Each suspension was then inoculated (1% *v*/*v*) with the adjusted overnight culture to yield a final starting bacterial concentration of approximately (1 × 10^6^ CFU/mL).

Cultures were incubated at 37 °C, in a 5% CO_2_ atmosphere for 24 h. After incubation, the number of viable bacteria in each culture was determined. Aliquots were serially diluted in phosphate-buffered saline, pH 7.0, and appropriate dilutions were plated on TSY agar (TSY broth solidified with 1.5% agar). Plates were incubated under the same conditions and the number of colonies was counted. Cultures without nanoparticles served as negative controls. To ensure high reproducibility, the entire evaluation was performed as two independent biological experiments conducted on separate time points. Within each independent experiment, six parallel replicates (sextuplicates) were evaluated for each condition. The results are presented as the average relative bacterial viability (%) compared to the negative control.

The antibacterial activity of the nanoparticles was also investigated against the anaerobic periodontal pathogen *Porphyromonas gingivalis* using the broth dilution method. A fresh culture of the *P. gingivalis* type strain DSM 20709 grown anaerobically in BM broth [22] was commercially purchased from the German Collection of Microorganisms and Cell Cultures (DSMZ, Braunschweig, Germany) [https://www.dsmz.de/collection/catalogue/details/culture/DSM-20709] (accessed on 15 June 2023) *Porphyromonas gingivalis* was diluted in broth to a final density of 10^6^ bacteria/mL. BM broth containing serially diluted concentrations of the various nanoparticle formulations (ranging from 0.125 to 0.5 mg/mL) was inoculated with 1% (*v*/*v*) of the bacterial suspension and incubated at 37 °C. Cultures were visually inspected daily for up to 24 h to determine the minimum inhibitory concentration (MIC), defined as the lowest concentration that fully prevented visible growth. The MIC endpoints were independently verified by two operators to ensure objectivity. All experimental procedures were performed in an anaerobic chamber maintained at 5% CO_2_, 9% H_2_, and 86% N_2_.

### 2.7. Reactive Oxygen Species (ROS) and Total Antioxidant Activity (TAC) Assay

The bacterial and hPDLC suspensions described in the antibacterial and MTT assay sections were utilized to evaluate intracellular ROS and cellular TAC levels, respectively. Specifically, the cell-permeable ROS-sensitive probe 2′,7′-dichlorodihydrofluorescein diacetate (CM-H2DCFDA), which fluoresces at 520 nm (ex = 480 nm) after oxidation, was used to quantify the levels of intracellular ROS and the TEAC technique was applied to evaluate the TAC levels using a commercial colorimetric kit (TAC colorimetric assay kit, Cayman Chemical Co., Ann Arbor, MI, USA) [23].

### 2.8. Statistical Analysis

Statistical analysis was performed using Student’s *t*-test for predefined pairwise comparisons between experimental groups and their corresponding controls. The analysis was applied to evaluate targeted differences related to nanoparticle composition, extract loading, and concentration within each assay. Statistical significance was set at *p* < 0.05.

## 3. Results and Discussion

**Figure 2:**
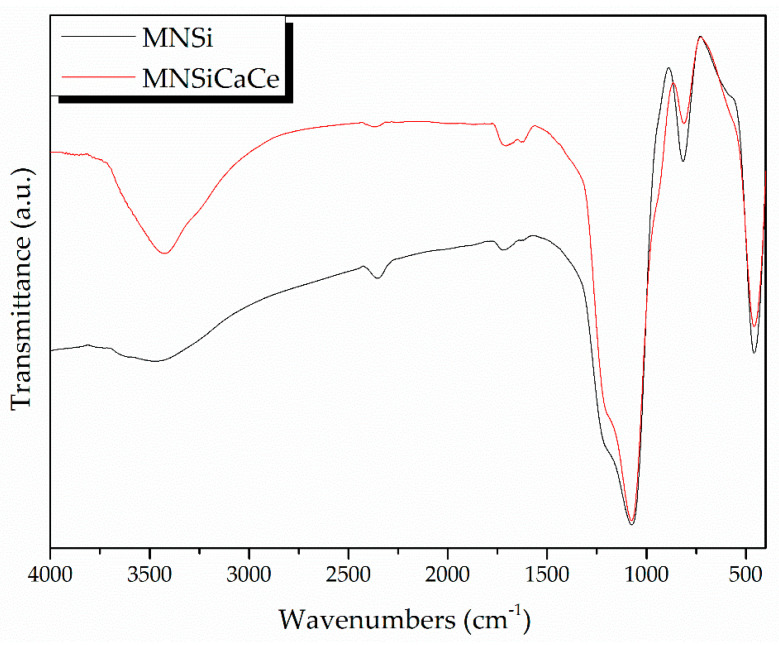
FTIR spectra of the synthesized nanoparticles.

FTIR analysis. FTIR spectra of synthesized silica nanoparticles (MNSi) and the MNSiCaCe NPs are presented in Figure 2. Prior to spectral interpretation, all FTIR spectra were baseline-corrected and normalized. The peak at about 1640 cm^−1^ is assigned to the vibration of hydrogen bonds between the Si–OH groups and the water molecules due to absorbed water. Stretching vibrations of the Si–OH bond were observed at the peak close to 963 cm^−1^. This peak disappears in the spectra of the samples containing calcium and cerium and is replaced by a shoulder that broadens the region between 1300–950 cm^−1^, which can be assigned to the formation of Si–O–Ca and Si–O–Ce bonds [24,25].

The broad band between 1300 and 950 cm^−1^ represents the asymmetric stretching vibrations of the bonds of the Si–O–Si group, while the bending vibrations of these bonds are observed at the peak at 798 cm^−1^. The decreased in intensity of the peak at 798 cm^−1^ after Ca and Ce doping, may be attributed to some loosening in the glassy network caused by the addition of dopant ions to the silica host matrix. This may be due to the breaking action of Si–O–Si (bridging oxygens) and the formation of non-bridging oxygen groups as Si-O-NBO by the dopant ions [26].

The incorporation of cations such as Ca^2+^ and Ce^3+^ into the open silica network disturbed the SiO_x_ tetrahedral network connectivity. This phenomenon is evidenced by the reduction in intensity and broadening of the shoulder around 975 cm^−1^. Additionally, the structural changes in doped silica samples are confirmed by the C–O bond vibrations observed in the 1420–1500 cm^−1^ range. These observations further confirm that Ca and Ce ions act as network modifiers, which enhance the content of non-bridging oxygen and change the silica network structure [20].

Z-potential. The nanoparticles exhibited a zeta potential of −29.4 ± 1.4 mV (mean ± standard deviation). The negative surface charge is consistent with the presence of deprotonated silanol groups on the silica surface and may help prevent particle aggregation through electrostatic repulsion.

**Figure 3:**
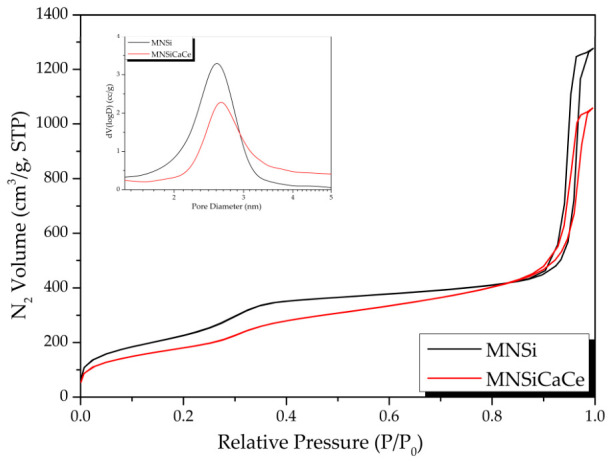
Nitrogen adsorption–desorption isotherms of MSNs and their associated pore size distribution.

BET and BJH analyses. Nitrogen adsorption–desorption measurements were performed to investigate the textural properties of the MNSi and MNSiCaCe nanoparticles (Figure 3). According to the IUPAC classification, the MNSi isotherm corresponds to a Type IVb isotherm, which is characteristic of mesoporous materials. The isotherm presents three distinct regions: (i) micropore filling at relative pressures (P/P_0_) below 0.05, (ii) capillary condensation in mesopores within the range 0.2 < P/P_0_ < 0.4, and (iii) condensation in interparticle voids at P/P_0_ > 0.9. Notably, the absence of hysteresis indicates the presence of tubular mesopores with diameters smaller than 4 nm in MNSi [20,24].

In contrast, the MNSiCaCe nanoparticles exhibit a Type II isotherm accompanied by a hysteresis loop of mixed H3 and H4 types. This observation suggests a potential shift from mesoporosity to microporosity, possibly due to pore blocking or partial filling upon the incorporation of calcium and cerium. H3 hysteresis is typically associated with slit-shaped pores or layered structures, while H4 hysteresis is more characteristic of mesoporous materials with narrow pore size distributions. Compared to the plain MSNs, the MSN-CaCe nanoparticles exhibit a reduced specific surface area accompanied by an increased average pore size. This trend likely reflects the partial occlusion of pore space and/or alterations to the mesoporous structural integrity caused by the introduction of Ca and Ce ions. The pore size distribution of MNSi and MNSiCaCe nanoparticles shows a porosity profile of around 2 nm attributed to the intrinsic mesoporous channels of the silica framework. MNSiCaCe distribution is broader and attenuated than MNSi, indicating that the introduction of Ca and Ce slightly degrades the organization of the silica network and increases the heterogeneity of the pores. Overall, these results provide evidence that doping modifies both the surface characteristics and pore architecture of mesoporous silica nanoparticles [24,27].

**Figure 4:**
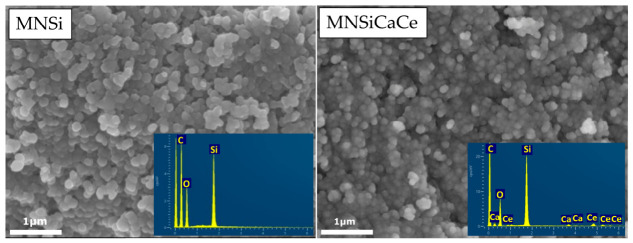
Representative SEM micrographs of mesoporous nanoparticles. Magnification ×20,000.

SEM analysis. MNSi and MNSiCaCe nanoparticles both exhibited a predominantly spherical morphology, nanoscale dimensions, and uniform distribution (Figure 4). The particle size of MNSi ranged from 24 nm to 150 nm. The MNSiCaCe nanoparticles displayed comparable shape characteristics but showed slightly increased aggregation, likely attributed to the incorporation of calcium and cerium ions. Despite this minor aggregation, the doping did not significantly alter the structural integrity or nanoscale size of the particles, as evidenced by the consistent morphology and size distribution observed in both samples.

### Artemisinin Loading Capacity and Controlled-Release Kinetics of Modified Mesoporous Silica Nanoparticles

**Figure 5:**
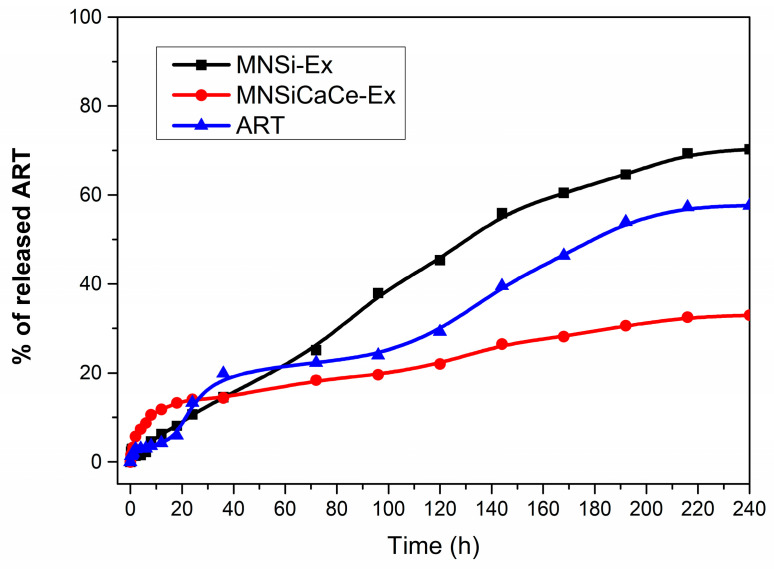
Cumulative artemisinin release (%) from MNSi-Ex and MNSiCaCe-Ex nanoparticles over 240 h. Free artemisinin (ART) was included as a control.

The artemisinin loading capacity of the synthesized mesoporous silica nanoparticles, along with their subsequent in vitro artemisinin (ART) release kinetics over a 240 h period, are integrated into a comprehensive profile (Figure 5 and Table 2). Quantitative analysis revealed a remarkable difference in drug loading capacity between the two carriers: the unmodified MNSi nanoparticles exhibited a loading capacity of 16.96%, whereas the multi-substituted MNSiCaCe matrix demonstrated a significantly higher loading capacity of 43.11%. Despite its substantially higher initial loading, the MNSiCaCe framework achieved a much more controlled and restricted release profile over time. All evaluated formulations displayed a characteristic two-phase release curve, initiating with a burst release phase during the first 24 h followed by a sustained-release plateau. The MNSi-Ex nanoparticles, characterized by a lower initial drug payload, delivered the highest relative cumulative release, exceeding the free-drug release profile at 40 h to reach 70% by the end of the study. Conversely, the MNSiCaCe-Ex system, which successfully loaded nearly triple the amount of artemisinin compared to MNSi, suppressed long-term diffusion significantly. After a slight initial burst phase (~13% in the first 20 h), its release curve plateaued after 40 h, resulting in a final cumulative release of just 33% at 240 h. Taken together, these results demonstrate that while the incorporation of calcium and cerium into the silica matrix markedly enhances the framework’s overall loading capacity for artemisinin, it simultaneously introduces dense structural modifications or stronger chemical retention, yielding a highly prolonged, slow-release therapeutic system.

*A. absinthium* extract phytochemical profiling. The total phenolic content of *Artemisia absinthium* extract was determined to be 182 ± 3.6 mg GAE per 100 g of dry plant material, while the total flavonoid content reached 42.5 ± 0.6 mg QE per 100 g dry plant material. The antimicrobial activity of the extract reflects the combined effects of multiple polyphenolic or other compounds. While identifying the individual contribution of each specific component was beyond the scope of this study, an estimation of the content of artemisinin and four polyphenolic compounds with known antimicrobial action was attempted based on their chromatographic profile and the comparison with analytical standards [28,29,30,31]. In the present extract, artemisinin was detected at a concentration of 3.77 mg/100 g dry plant material. Gallic acid was detected at 5.5 ± 0.7 mg/100 g dry plant material (RT = 6.901 min), while methyl caffeate was found at 4.2 ± 0.3 mg/100 g dry plant material (RT = 22.906 min). Notably, astragalin was the predominant compound, reaching 37.0 ± 1.1 mg/100 g dry plant material (RT = 24.749 min), whereas quercetin was present at 4.2 ± 0.8 mg/100 g dry plant material (RT = 30.087 min). Representative HPLC chromatograms are provided in Supplementary Figure S1, showing the identified peaks of gallic acid, methyl caffeate, astragalin, quercetin, and artemisinin.

**Figure 6:**
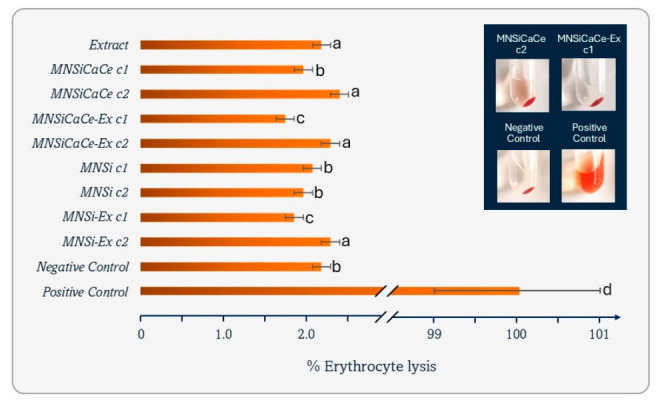
Relative (%) erythrocyte lysis following co-incubation with mesoporous silica nanoparticles (MNSi) and calcium- and cerium-doped variants (MNSiCaCe). Selected formulations were loaded with *A. absinthium* extract (-Ex). Two concentrations (c1: 0.125 and c2: 0.5 mg/mL) were tested for each formulation. The insert displays representative reaction tubes: intact erythrocytes form a pellet at the bottom after centrifugation (negative control), whereas lysis results in a red-colored supernatant with no visible pellet, similar to the positive control. Different letters indicate statistically significant differences among the different groups from pairwise comparisons performed within the same experiment.

Hemocompatibility and MTT assay evaluation. All nanoparticle preparations exhibited minimal hemolytic activity, remaining below 2%, comparable to the negative control (Figure 6). The positive control exhibited complete hemolysis, approaching 100%, thus confirming the sensitivity and reliability of the assay. These findings confirm the exceptional hemocompatibility of the nanoparticles, satisfying a critical prerequisite for biomaterials intended for systemic or dental applications.

Comparable hemocompatibility was previously reported by our group for ART-loaded mesoporous silica and cerium-doped nanoparticles [32]. No adverse interactions of those nanoparticles were found with blood components. Specifically, MCM-41-type MSNs with particle sizes approximately 100 nm were shown to adhere to red blood cell surfaces without compromising membrane integrity or morphology. Taken together, these collective findings reinforce the suitability of silica-based mesoporous nanoparticles for applications involving direct contact with the circulatory system. In a related study, composite coatings of chitosan and mesoporous bioactive glass nanoparticles loaded with gentamicin were applied onto titanium substrates via electrophoretic deposition. These coatings exhibited enhanced hemocompatibility and antibacterial performance, indicating potential for improving implant integration and minimizing infection risk [33]. Building on these insights, the present work further explores the functionalization of mesoporous systems to enhance both biological safety and therapeutic performance in tissue regeneration applications [34,35].

**Figure 7:**
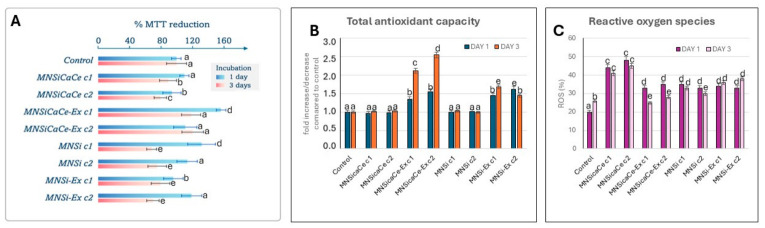
(**A**) Viability, expressed as % MTT reduction, of human periodontal ligament cells after co-incubation with mesoporous silica nanoparticles (MNSi) and calcium- and cerium-doped (MNSiCaCe) for 1 and 3 days. Some formulations were loaded with *A. absinthium* extract (-Ex). Two concentrations (c1: 0.125 and c2: 0.5 mg/mL) of all formulations were tested. (**B**) Total antioxidant capacity of hPDLCs cultured with the same conditions as those used for the MTT assay and are expressed in fold adjustment compared to the control group (cells without NPs). (**C**) Intracellular ROS levels of hPDLCs at the same conditions. Different letters indicate statistically significant differences among the different groups from pairwise comparisons performed within the same experiment.

The results of the MTT assay indicate that, after 24 h of incubation, all tested materials exhibited increased enzymatic activity compared to the control group (Figure 7). Notably, hPDLCs cultured with the lower concentration of MNSi nanoparticles showed the highest MTT reduction percentage of amount among the undoped samples at 24 h. Among all groups, the extract-loaded MNSiCaCe nanoparticles at a concentration of 0.125 mg/mL demonstrated the most pronounced increase in MTT reduction, reaching nearly 160% relative to control cells. After 3 days of incubation, a slight reduction in hPDL viability was observed in most groups compared to the control. However, the MNSiCaCe nanoparticles with *A. absinthium* extract at both concentrations continued to promote cell viability, reaching up to 120% MTT reduction compared to the control (Figure 7). These findings suggest a positive synergistic effect between *A. absinthium* extract and the MNSiCaCe nanoparticles. This enhancement may be attributed to the coordinated release of phytochemical constituents of the *Artemisia* extract and metal ions, which appears critical for the observed biological activity. The influence of *A. absinthium* extract on hPDL proliferation is an emerging area of interest in stem cell research, particularly for applications in tissue regeneration and periodontal therapy. Although direct investigations into the interactions between this specific extract and hPDLCs remain limited, recent work by our group demonstrated that *A. absinthium* extract alone was not cytotoxic to hPDL. Instead, it enhanced cell metabolic activity after 3 days of exposure [17,36]. Moreover, extracts from *Artemisia* species, including artemisinin-containing preparations, have demonstrated excellent biocompatibility in previous studies [37]. These extracts have shown low cytotoxicity toward various mammalian cell lines at therapeutic concentrations [38]. Their favorable safety profile supports their use in biomedical applications, including wound healing and tissue regeneration. The capacity of *Artemisia* extracts to promote cell viability and reduce inflammation further reinforces their potential as natural bioactive agents in biomaterials and drug delivery systems [39].

**Figure 8:**
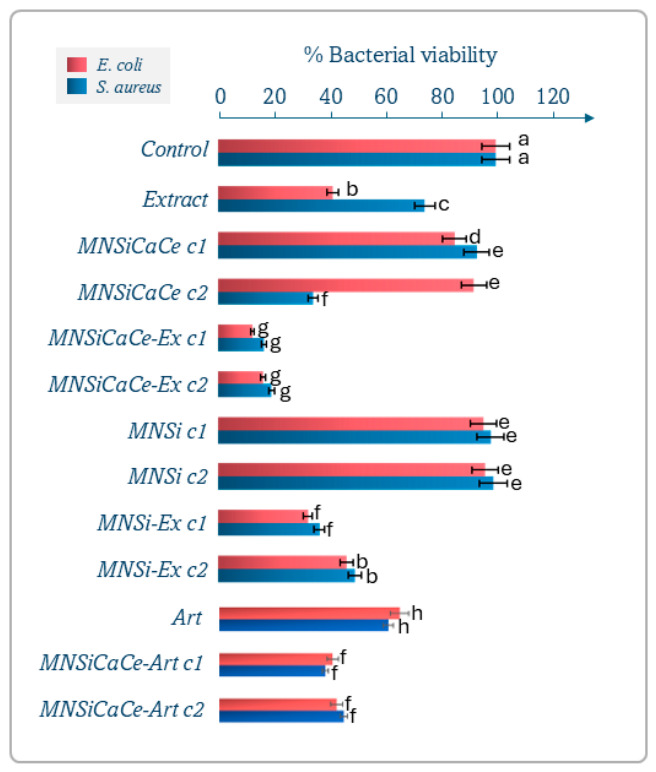
Bacterial viability of *S. aureus* and *E. coli* incubated in the presence of Art (artemisinin), *A. absinthium* extract, mesoporous silica nanoparticles (MNSi), and calcium- and cerium-doped nanoparticles (MNSiCaCe). Some nanoparticle formulations were loaded with *A. absinthium* extract (-Ex) (blue) and ART (deep blue). Two concentrations (c1: 0.125 and c2: 0.5 mg/mL) of all formulations were tested. Viability was estimated by the number of colony forming units/mL detected by cultivation of each sample. It is expressed in % of the control. Different letters indicate statistically significant differences (*p* < 0.001) between the tested groups.

Antibacterial activity. Figure 8 illustrates the antibacterial activity of various nanoparticles against *E. coli* and *S. aureus*, as reflected by their impact on bacterial viability. *A. absinthium* extract alone significantly reduced bacterial viability, with *S. aureus* exhibiting greater sensitivity to the extract than *E. coli*. MNSiCaCe nanoparticles demonstrated concentration-dependent antibacterial activity against the Gram-positive *S. aureus*, with the effect being more pronounced at the higher concentration.

When the extract was combined with MNSiCaCe nanoparticles, the antibacterial efficacy was enhanced for both bacterial species, reducing viability to below 20% indicating a possible synergistic interaction. In contrast, MNSi nanoparticles lacking cerium and calcium at both concentrations showed no antibacterial effect when used alone. Nevertheless, their activity improved upon combination with extract, although the effect remained less potent than that observed for the MNSiCaCe-based formulations. The stronger susceptibility of *S. aureus* (Gram-positive) compared with *E. coli* (Gram-negative) is likely associated with fundamental differences in bacterial cell envelope organization. The outer membrane which functions as an effective permeability barrier that restricts nanoparticle penetration and limits the diffusion of hydrophobic phytochemicals or metal ions into the cell. Consequently, Gram-negative *E. coli* generally exhibits greater intrinsic resistance to nanomaterials and plant-derived antimicrobials than Gram-positive bacteria, whose more exposed peptidoglycan matrix allows closer interaction with nanoparticle surfaces and facilitates membrane disruption, oxidative stress, and intracellular damage [40,41].

The enhanced antibacterial efficacy observed for the combination of *A. absinthium* extract with MNSiCaCe nanoparticles is consistent with a possible synergistic interaction. Phytochemicals present in the extract, including flavonoids and phenolic acids, may destabilize bacterial membranes, increase membrane permeability, chelate essential metals, or interfere with metabolic enzymes, thereby potentially sensitizing bacterial cells to nanoparticle-induced stress. Simultaneously, the nanoparticles may facilitate adsorption, stabilization, or localized delivery of these bioactive compounds onto the bacterial surface, increasing their effective concentration at the site of action. Such complementary mechanisms frequently result in stronger antibacterial responses than either component used alone [42]. Furthermore, evaluating pure artemisinin (ART) clarifies its role as an important active driver behind the antimicrobial action of the *A. absinthium* extract. Specifically, the crude extract demonstrated a clear preference for the Gram-negative *E. coli*, reducing its viability much more effectively (~42%) than that of the Gram-positive *S. aureus* (~73%), whereas pure ART maintained a relatively uniform, moderate efficacy against both strains (~60–65% viability). Interestingly, when integrated into the nanoparticles (*MNSiCaCe-Art c1* and *c2*), artemisinin successfully maintained a steady, controlled reduction in viability down to approximately 40–50% for both *E. coli* and *S. aureus*. While these ART-loaded nanoparticles did not achieve the most potent bactericidal efficacy, the extract-loaded matrix (MNSiCaCe-Ex), the observed antimicrobial activity of ART supports its contribution as one of the functional components of the extract. This indicates that the enhanced activity observed in the MNSiCaCe-Ex formulations is heavily related to artemisinin, together with complementary secondary metabolites present in the crude extract.

A further explanation of the synergistic effect observed for the loaded NPs with ART and extract, respectively, may involve a redox recycling mechanism associated with cerium-containing nanoparticles. Cerium oxide-based systems can reversibly cycle between Ce^3+^ and Ce^4+^ oxidation states through oxygen vacancy-mediated surface reactions, enabling repeated catalytic redox activity. In the presence of reducing phytochemicals from the extract, oxidized Ce^4+^ centers may be converted back to Ce^3+^, for the loaded NPs with ART and extract, respectively, at the nanoparticle–bacteria interface, consistent with the elevated ROS levels observed in the present study. This regenerative redox cycling may intensify oxidative damage to bacterial membranes, proteins, and nucleic acids, thereby contributing to the markedly reduced viability observed in the combined treatments. Calcium incorporation may additionally influence surface charge, ionic interactions, structural defects, or particle dispersion, further promoting bacterial contact and catalytic efficiency [43]. Furthermore, the marked variance in the susceptibility of Gram-negative *E. coli* (~42% viability) and Gram-positive *S. aureus* (~73% viability) to the unencapsulated crude extract can be attributed to the fundamental differences in their cellular wall architecture and lipid membrane composition. Gram-negative bacteria like *E. coli* possess a distinct outer lipid membrane primarily composed of lipopolysaccharides (LPS) and phospholipids, which surrounds a relatively thin layer of peptidoglycan. Crude plant extracts from the *Artemisia* genus contain a complex mixture of hydrophobic secondary metabolites, such as volatile terpenes, sesquiterpenes, and phenolic compounds. These lipophilic constituents can interact favorably with, partition into, and disrupt the outer lipid bilayer of *E. coli*, leading to increased membrane permeability, loss of intracellular components, and a subsequent decline in cell viability.

The MIC values of extract and the various nanoparticle formulations for *P. gingivalis* are shown in Table 3. The extract encapsulated within the doped nanoparticles exhibited the lowest MIC value compared with the other tested materials.

Overall, these findings highlight the potential of combining *A. absinthium* extract with cerium- and calcium-doped mesoporous silica nanoparticles to achieve enhanced antibacterial activity against both *S. aureus* and *E. coli*. This is supported by previous studies demonstrating the potent antibacterial properties of artemisinin and related compounds, which have been shown to effectively inhibit bacterial growth by inducing oxidative stress and compromising bacterial membrane integrity [44].

**Figure 9:**
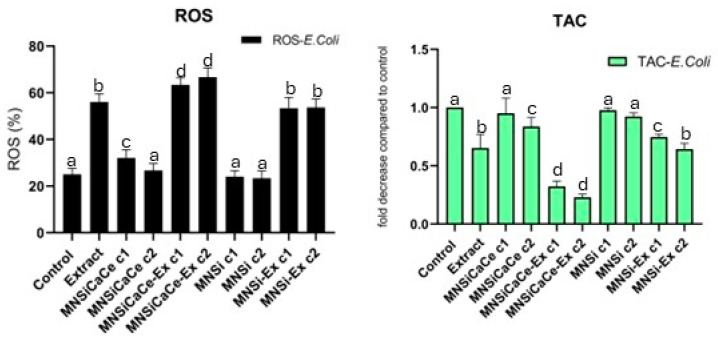
ROS levels and TAC fold increase compared to control of *E. coli* incubated in the presence of *A. absinthium* extract, mesoporous silica nanoparticles (MNSi), and calcium- and cerium-doped nanoparticles (MNSiCaCe). Some nanoparticle formulations were loaded with *A. absinthium* extract (-Ex). Two concentrations (c1: 0.125 and c2: 0.5 mg/mL) of all formulations were tested. Different letters indicate statistically significant differences (*p* < 0.001) between the tested groups.

ROS and TAC levels. Figure 9 presents the oxidative stress biomarkers after 24 h of incubation of the tested material with *E. coli* as previously described. ROS levels increased markedly in cells treated with MNsiCace and MNsiCace-Ex at both concentrations, indicating enhanced oxidative stress, while the extract alone and MNSi treatments resulted in comparatively lower ROS levels, closer to the control. Conversely, cellular TAC profiles demonstrated a strong inverse correlation with ROS generation, specifically, treatments that induced higher ROS were associated with a pronounced reduction in antioxidant capacity, particularly MNsiCace-Ex, which showed the greatest decrease relative to the control. Conversely, the extract and MNSi treatments maintained TAC values closer to control levels, suggesting better preservation of antioxidant defenses. Together, these results indicate that MNsiCace-based treatments promote oxidative stress in *E. coli* by increasing ROS while simultaneously reducing total antioxidant capacity.

## 4. Conclusions

The newly synthesized mesoporous silica-based nanoparticles exhibited predominantly spherical morphology, nanoscale dimensions, and characteristic porous architecture. The MNSiCaCe nanoparticles showed higher artemisinin loading capacity and a more sustained release profile compared with undoped MNSi, indicating their potential as controlled-delivery carriers for *A. absinthium* extract components. The materials demonstrated low hemolytic activity and favorable in vitro compatibility with human periodontal ligament cells, particularly when MNSiCaCe nanoparticles were loaded with *A. absinthium* extract. Furthermore, strong antibacterial activity was observed, particularly when the extract was combined with the doped nanoparticles, which was mediated by a dual mechanism of action involving intracellular ROS generation and the concomitant exhaustion of antioxidant capacity in bacterial cells, effectively reducing bacterial viability. These findings support MNSiCaCe nanoparticles as promising in vitro nanocarriers for enhancing the antibacterial activity of natural bioactive compounds. Further studies, including extended mechanistic assays, ion-release analysis, bactericidal testing, and in vivo evaluation, are required to confirm their therapeutic potential.

## Figures and Tables

**Table 1:** Nominal compositions of the obtained silica-based mesoporous NPs in % mol.

Sample Name	SiO_2_	CaO	CeO
MNSi	100		
MNSiCaCe	60	35	5

**Table 2:** Loading capacity of NPs after 24 h of incubation with *Artemisia absinthium* extract containing artemisinin.

Sample	Loading Capacity (%)
MNSi	16.96
MNSiCaCe	43.11

**Table 3:** MIC values of the extract and the tested materials.

Compounds	Minimal Inhibitory Concentration (MIC)
A/E	2.5 μg/mL
ART	42 μM
MNSiCaCe	>0.5 mg/mL
MNSiCaCe-A/E (2.5 µg/mL)	>0.125 mg/mL
MNSiCaCe-Art	>0.250 mg/mL
MNSi	>0.5 mg/mL

A/E: *Artemisia* extract.

## Data Availability

The original contributions presented in this study are included in the article. Further inquiries can be directed to the corresponding author.

## Supplementary Materials

The following supporting information can be downloaded at https://www.mdpi.com/article/10.3390/jfb17070326/s1. Supplementary Figure S1. Chromatograms recorded at (A) 275 nm, (B) 325 nm, and (C) 210 nm, respectively. Arrows indicate the peaks corresponding to gallic acid (1), methyl caffeate (2), astragalin (3), quercetin (4), and artemisinin (5).
