# Supplementary material for: Enhanced Antibacterial Activity of Artemisia absinthium Extract Containing Artemisinin and Polyphenols Loaded into Mesoporous Silica Calcium- and Cerium-Doped Nanoparticles
Source: J Funct Biomater. 2026 Jul 6;17(7):326. doi: 10.3390/jfb17070326 (PMC13413392; doi:10.3390/jfb17070326)
Supplement: Supplementary file 1 [file jfb-17-00326-s001.zip › jfb-4310376-supplementary.pdf]

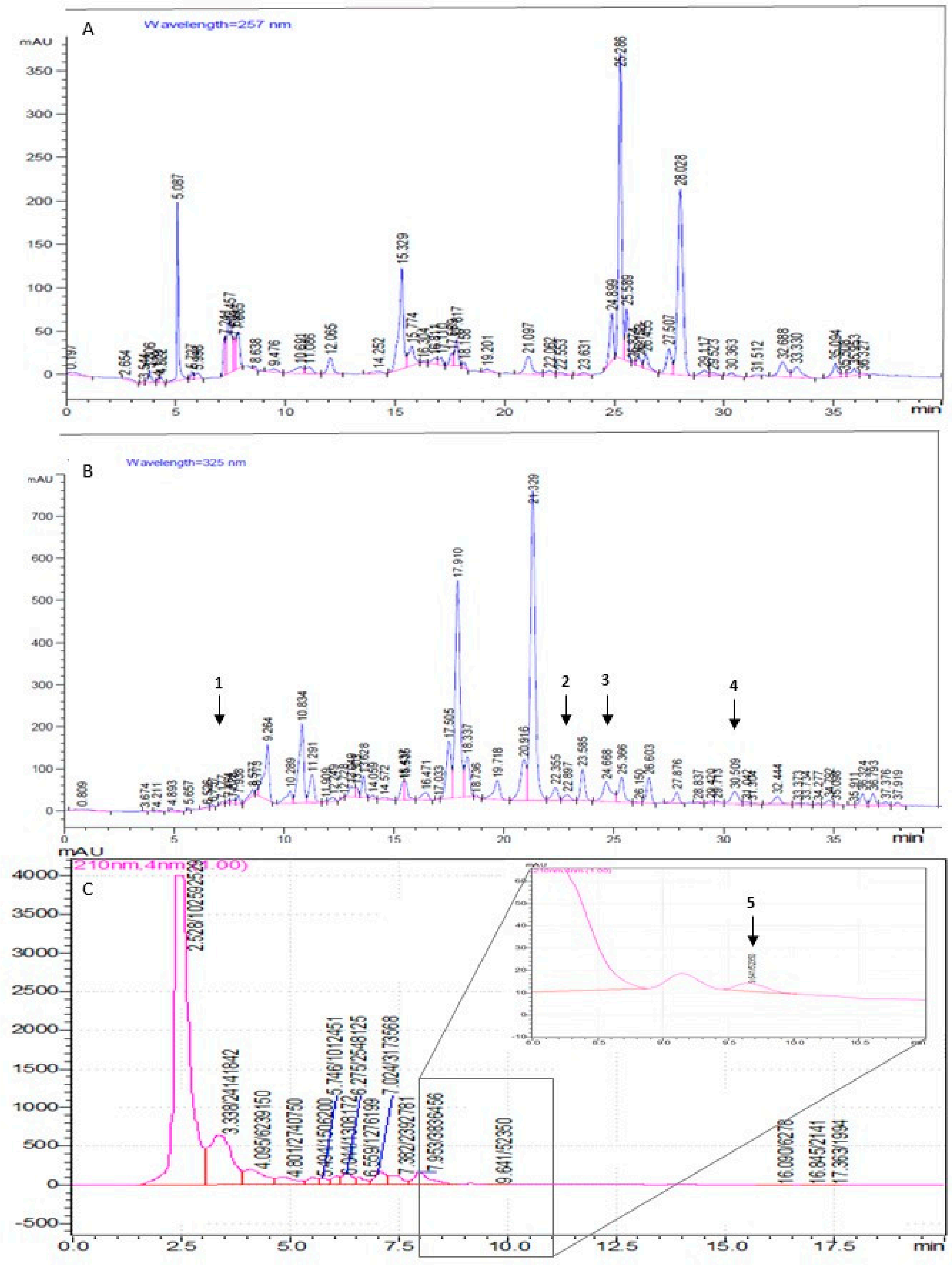

Supplementary Figure S1. Representative HPLC chromatograms recorded at (A) 275 nm, (B) 325 nm, and (C) 210 nm. Arrows indicate the peaks corresponding to gallic acid (1), methyl caffeate (2), astragalin (3), quercetin (4), and artemisinin (5).
